# Antibody Response to SARS-CoV-2 Infection and Vaccination in COVID-19-naïve and Experienced Individuals

**DOI:** 10.3390/v14020370

**Published:** 2022-02-10

**Authors:** Susan L. Uprichard, Amornrat O’Brien, Monika Evdokimova, Cynthia L. Rowe, Cara Joyce, Matthew Hackbart, Yazmin E. Cruz-Pulido, Courtney A. Cohen, Michelle L. Rock, John M. Dye, Paul Kuehnert, Keersten M. Ricks, Marybeth Casper, Lori Linhart, Katrina Anderson, Laura Kirk, Jack A. Maggiore, Andrew S. Herbert, Nina M. Clark, Gail E. Reid, Susan C. Baker

**Affiliations:** 1 Department of Medicine, Stritch School of Medicine, Loyola University Chicago, Maywood, IL 60153, USA; MBCASPER@lumc.edu (M.C.); loril417@gmail.com (L.L.); kaanderson@luhs.org (K.A.); r-kirk@att.net (L.K.); NMCLARK@lumc.edu (N.M.C.); greid@lumc.edu (G.E.R.); 2 Department of Microbiology and Immunology, Stritch School of Medicine, Loyola University Chicago, Maywood, IL 60153, USA; aobrien6@luc.edu (A.O.); mevdokimova@luc.edu (M.E.); cynthiarowe@comcast.net (C.L.R.); hackbart@wustl.edu (M.H.); ycruz@luc.edu (Y.E.C.-P.); sbaker1@luc.edu (S.C.B.); 3 Infectious Disease and Immunology Research Institute, Stritch School of Medicine, Loyola University Chicago, Maywood, IL 60153, USA; 4 Department of Public Health Sciences, Parkinson School of Health Sciences and Public Health, Loyola University Chicago, Maywood, IL 60153, USA; cjoyce6@luc.edu; 5 Viral Immunology Branch, Virology Division, United States Army Medical Research Institute of Infectious Diseases, Fort Detrick, Frederick, MD 21702, USA; courtney.a.cohen.ctr@mail.mil (C.A.C.); michelle.l.rock5.ctr@mail.mil (M.L.R.); john.m.dye1.civ@mail.mil (J.M.D.); andrew.s.herbert4.civ@mail.mil (A.S.H.); 6 The Geneva Foundation, Tacoma, WA 98042, USA; 7 Diagnostic Systems Division, United States Army Medical Research Institute of Infectious Diseases (USAMRIID), Frederick, MD 21702, USA; paul.a.kuehnert.mil@mail.mil (P.K.); keersten.m.ricks.civ@mail.mil (K.M.R.); 8 Department of Pathology, Stritch School of Medicine, Loyola University Chicago, Maywood, IL 60153, USA; Jack.Maggiore@lumc.edu

**Keywords:** SARS-CoV-2, COVID-19, vaccine response, antibodies, mRNA-1273, BNT162B2

## Abstract

Understanding the magnitude of responses to vaccination during the ongoing SARS-CoV-2 pandemic is essential for ultimate mitigation of the disease. Here, we describe a cohort of 102 subjects (70 COVID-19-naïve, 32 COVID-19-experienced) who received two doses of one of the mRNA vaccines (BNT162b2 (Pfizer–BioNTech) and mRNA-1273 (Moderna)). We document that a single exposure to antigen via infection or vaccination induces a variable antibody response which is affected by age, gender, race, and co-morbidities. In response to a second antigen dose, both COVID-19-naïve and experienced subjects exhibited elevated levels of anti-spike and SARS-CoV-2 neutralizing activity; however, COVID-19-experienced individuals achieved higher antibody levels and neutralization activity as a group. The COVID-19-experienced subjects exhibited no significant increase in antibody or neutralization titer in response to the second vaccine dose (i.e., third antigen exposure). Finally, we found that COVID-19-naïve individuals who received the Moderna vaccine exhibited a more robust boost response to the second vaccine dose (*p* = 0.004) as compared to the response to Pfizer–BioNTech. Ongoing studies with this cohort will continue to contribute to our understanding of the range and durability of responses to SARS-CoV-2 mRNA vaccines.

## 1. Introduction

Severe acute respiratory syndrome coronavirus 2 (SARS-CoV-2) is a single-stranded RNA betacoronavirus that emerged in 2019 and is the causative agent of the ongoing coronavirus disease 2019 (COVID-19) pandemic [1,2]. Outcomes after infection range from asymptomatic to severe disease to death, resulting in millions of deaths worldwide [3]. The genome of SARS-CoV-2 encodes four structural proteins, a nucleocapsid (N) protein surrounded by an envelope containing three membrane proteins: membrane (M), envelope (E), and spike (S), which is divided into two functional subunits, S1 and S2 [4]. Of these proteins, S and N elicit robust adaptive immune responses and have been widely used to detect seroconversion after infection [5,6].

The receptor-binding domain (RBD) within the S1 subunit of the viral spike protein directly interacts with the cellular angiotensin-converting enzyme 2 (ACE2) receptor to mediate host cell entry [7,8,9,10,11]. By binding to the RBD, antibodies can block the attachment of the virus to ACE2 and neutralize the virus [12]. Thus, analyses of antibodies against spike often focus on the RBD. However, other regions within spike, such as the N-terminal domain (NTD) and S2 domain, also contain neutralizing epitopes [13,14,15,16,17], suggesting that evaluating the antibody response to the full spike protein and determining neutralizing titers provides a more comprehensive picture of the antibody response to natural infection and vaccination.

Virus-specific antibodies against the main viral immunogens, S and N, can be detected in most patients after SARS-CoV-2 infection [18,19,20]. While challenge studies conducted for seasonal coronaviruses demonstrate reasonably high levels of the baseline neutralizing antibody, protection from endemic coronavirus infection is short-lived [21,22,23]. Likewise, emerging data suggest a relatively rapid decline in SARS-CoV-2 antibodies post-infection [24,25,26,27,28]. Importantly, data from cell culture [13] and passive transfer of antibodies in non-human primates [29] indicate that circulating spike-specific antibodies provide protection against SARS-CoV-2 infection. Less than a year after COVID-19 was declared a pandemic, two mRNA vaccines, BNT162b2 (Pfizer–BioNTech) and mRNA-1273 (Moderna), received emergency use authorization in the U.S. based on data from initial trials indicating 95% protection from severe disease by both mRNA vaccines [30,31]. However, data are still emerging detailing the relative levels of immunity elicited by these SARS-CoV-2 vaccines, as they only became available for distribution in December 2020 [32,33].

If maintained at sufficiently high levels, antibodies induced by SARS-CoV-2 infection and/or vaccination should help block or attenuate infection and help end the pandemic. Because vaccination is the preferred/safer path to reach herd immunity [34,35], additional data independent from the original vaccine manufacturers’ selected populations is needed to further our understanding of the development and durability of virus-specific antibodies after SARS-CoV-2 vaccination. This knowledge will enable management of the pandemic, provide insight into the most effective vaccination practices (e.g., optimal administration of booster immunizations), and provide information regarding the utility of post-vaccination antibody testing.

To add to the critical mass of growing knowledge needed regarding the immune response to SARS-CoV-2 infection and vaccination, we established a longitudinal cohort of over 1000 individuals to follow serologically during the pandemic. Here we report on 70 SARS-CoV-2-naïve and 32 SARS-CoV-2-recovered individuals (“COVID-19-experienced”) who received two doses of one of the SARS-CoV-2 mRNA vaccines and provided serial blood samples. Using these longitudinal samples, we assessed serum antibodies prior to and after the first and second immunization doses, correlated this with SARS-CoV-2 neutralization, and assessed the impact of demographic variables on vaccine responses.

## 2. Materials and Methods

### 2.1. Study Design and Recruitment

Between June 2020–April 2021, 102 individuals 18 years of age or older (70 COVID-19-naïve, 32 COVID-19-experienced) gave written informed consent to participate in this prospective study, which was approved by the Loyola University Chicago Institutional Review Board (IRB# 213447032320 and 214521021621). Subjects were defined as COVID-19-naïve or COVID-19-experienced based on three criteria: self-reported history of a SARS-CoV-2-positive test, pre-existing antibodies against spike, and/or pre-existing antibodies against nucleocapsid. All subjects received either Pfizer (BNT162b2) or Moderna (mRNA-1273) mRNA vaccines. Peripheral venous blood and demographic/clinical questionnaire data were collected at three study visits: baseline (PV), 3 weeks post-initial vaccine dose (V1), and 3 weeks after the second vaccine (V2). Cohort demographics are provided in Table 1.

### 2.2. Quantitative Enzyme-Linked Immunosorbent Assay (ELISA)

ELISA was performed using the spike antigen (hexapro), which contains six point mutations that stabilize the extracellular domain of spike in its prefusion conformation [36]. The six histidine residue (6XHis)-tagged hexapro antigen was produced by the transient transfection of HEK-293T cells with the plasmid SARS-CoV-2 S HexaPro encoding the hexapro antigen (Addgene, Watertown, MA, USA, Cat#154754) using Mirus Trans LT1 (Mirus, Marietta, GA, USA, Cat# MIR 2305). The hexapro protein was purified by affinity chromatography utilizing Ni-NTA agarose (ThermoFisher Scientific, Waltham, MA, USA), and purity was assessed by sodium dodecyl sulfate polyacrylamide gel electrophoresis (SDS-PAGE). The ELISA was performed according to the method described by Stadlebauer et al. [37]. Donor sera were initially screened at a 1:450 dilution and compared with pre-pandemic controls to identify anti-SARS-CoV-2 spike responses. Antibody end-point titers were then determined by serial dilution of the samples to negativity to identify the point where the background subtracted absorbance was no longer distinguishable from the OD of the pre-pandemic negative control serums at the same dilution.

### 2.3. Magpix Multiplex Immunoassay

Multiplex immunoassays were performed as previously described (Johnston et al., 2021; Golden et al., 2021). The antigens for the assay were: recombinant SARS-CoV-2 full trimeric spike (gift from Dr. Jason McLellan’s group; UT-Austin [9]), S1 (Sino Biological, 40591-V08H, Chesterbrook, PA, USA), RBD (Sino Biological, 40592-V08H), and nucleocapsid protein (NP) (Native Antigen Company, REC31812-100, Kidlington, UK). Recombinant proteins were conjugated to magnetic microspheres using the Luminex xMAP1antibody coupling kit (Luminex Inc., Austin, TX, USA) according to the manufacturer’s instructions at a final concentration of 4 ug antigen: 1 × 10^6^ microspheres, a concentration that has been shown to be optimal for IgG and IgM detection [38]. SARS-CoV-2 full spike, S1, RBD, and NP were coupled to Magplex microsphere regions #45, #55, #65, and #25, respectively, in order to facilitate multiplexing experiments. Beads were stored at 4 °C until further use. Donor serum samples were diluted 1:100 in 1× PBS containing 0.02% Tween-20 (Sigma, St. Louis, MO, USA) (PBST) and 5% skim milk (PBST-SK). Each individual antigen-coupled bead was mixed at a 1:1 ratio prior to diluting in PBST to 5 × 10^4^ microspheres/mL, and the mixture was added to the wells of a Costar polystyrene 96-well plate at 50 uL per well (2500 microspheres of each antigen bead set/well). The plate was placed on a magnetic plate separator (Luminex Corp., Austin, TX, USA), covered with foil, and microspheres were allowed to collect for 60 s. While still attached to the magnet, the buffer was removed from the plate by inverting. Then, 50 uL of diluted serum samples were added to appropriate wells. The plate was covered with a black, vinyl plate cover and incubated with shaking for 1 h at ambient temperature. The plate was washed three times with 100 uL of PBST for each wash, using the plate magnet to retain the Magplex microspheres in the wells. Liquid was discarded by inverting. Next, 50 uL of a 1:100 dilution of goat anti-human IgG phycoerythrin conjugate (Sigma, P9170) in PBST-SK was added to the wells. The plate was covered again with a black, vinyl plate sealer and incubated with shaking for 1 h at ambient temperature. After incubation, the plate was washed three times as detailed above, and the Magplex microspheres were resuspended in 100 uL of PBST for analysis on the Magpix instrument (Luminex Corp, Austin, TX, USA). Raw data were reported as median fluorescence intensity for each bead set in the multiplex.

### 2.4. Abbott SARS-CoV-2 Anti-N IgG Assay

The Abbott anti-N chemiluminescent microparticle immunoassay was run in the Loyola University Medical Center Clinical Laboratory to qualitatively detect anti-N IgG antibodies to the nucleocapsid protein of SARS-CoV-2. This assay is currently approved for use under the United States Food and Drug Administration’s emergency use authorization. The assay was performed on an Abbott Laboratories ARCHITECT™ *i*2000SR analyzer. The signal was measured in relative light units (RLU). A direct, proportional relationship is established between the amount of IgG antibodies to SARS-CoV-2 in the sample and the RLU detected by the system optics following an established assay calibration. This relationship is reflected in the calculated index, the signal-to-cutoff ratio (S/CO). The positivity threshold has been established as S/CO exceeding a ratio of 1.4. Based on this S/CO, the results herein are presented as negative or positive.

### 2.5. SARS-CoV-2 Microneutralization Assay

Serum samples were diluted 1:10 in cell culture media (MEM; Corning, Corning, NY, USA, 10-010) containing 2% fetal bovine serum (FBS) (GE Healthcare, Chicago, IL, USA) and diluted 3-fold for a 9-point dilution curve. Naïve human sera (Sigma, St. Louis, MO, USA, H4522) and SARS-CoV-2 convalescent sera were used as a negative and positive control, respectively. Diluted serum samples were mixed 1:1 with the SARS-CoV-2 WA1 strain and incubated at 37 °C for 1 h. The serum–virus mixture was then added to Vero E6 cells at a multiplicity of infection (MOI) of 0.2 and incubated at 37 °C for 1 h. Unbound virus was removed, fresh cell culture media (MEM/10% FBS/1% PenStrep) was added to each well, and the infection progressed for an additional 23 h. Cells were then fixed in 10% formalin, washed three times with PBS, permeabilized with 0.2% Triton X-100, and blocked with Cell Staining Buffer (BioLegend, San Diego, CA, USA, 420201). The number of infected cells was determined using SARS-CoV-nucleocapsid–specific monoclonal antibody (Sino Biological, 40143-R001) and goat anti-rabbit IgG (H + L) Alexa Fluor 488 fluorescently labeled secondary antibody (Life Technologies, Carlsbad, CA, USA, A11008). The percentage of infected cells was determined with an Operetta high-content imaging instrument, and data analysis was performed using the Harmony software (Perkin Elmer, Naperville, IL, USA). Percentage neutralization for each sample was determined relative to untreated, virus-only control wells.

### 2.6. Statistical Methods

Patient characteristics are presented overall and stratified based on previous COVID-19 infection status (experienced versus naïve), and group differences were assessed for statistical significance using a *t*-test for age and chi-square or Fisher’s exact test for nominal variables. To assess differences in immune response before vaccination, after the first vaccination dose, and after the second vaccination dose, the natural log of endpoint antibody titer was regressed on COVID-19 exposure, time-point, and the exposure by time interaction term while controlling for age, gender, and race using linear mixed-effects models (LMM). LMMs included random intercepts to account for correlation due to repeated participant measurements. Among the COVID-19-naïve only, the magnitude of vaccine response was compared by each participant characteristic using a separate LMM that included the characteristic of interest, time, and an interaction term. Adjusted means were plotted by participant characteristic and time-point. Correlations at primary exposure (CE PV and CN-V1) and first boost (CE-V1 and CN-V2) were estimated with Spearman’s rho. Analyses were performed using SAS 9.4 (SAS Institute, Cary, NC, USA).

## 3. Results

To assess antibody response to the two-dose SARS-CoV-2 mRNA vaccines (Pfizer BNT162b2 and Moderna mRNA-1273), we prospectively enrolled participants and collected longitudinal serum samples and data. Peripheral blood samples were collected for quantitative enzyme-linked immunosorbent assay (ELISA) of anti-spike antibodies pre- and post-vaccination, with a focus on three timepoints that allowed analysis of the immune responses following both primary and secondary immunizations, that is, pre-vaccine baseline (PV), 3 weeks following the first vaccine dose (V1) and 3 weeks following the second vaccine dose (V2). The mean age was 46 ± 13, and the majority were women (*n* = 77, 75.5%), Caucasian (*n* = 79, 77.5%), and healthcare workers (*n* = 88, 86.3%). Only 25% of participants self-reported any comorbidities, with the most prevalent comorbidity reported being cardiovascular disease (CVD)(*n* = 17, 16.7%) (Table 1).

### 3.1. Defining SARS-CoV-2 Infection Status Prior to Vaccination

Because a large percentage of SARS-CoV-2 infections is asymptomatic and tests were in short supply early in the pandemic [39], many of those enrolled in our study may have been infected, but not tested. Therefore, participants’ prior infection status was determined using three indications of infection: a self-reported positive RT-qPCR test, detection of antibodies against the viral nucleocapsid protein (anti-N), and/or detection of pre-vaccination antibodies against the viral spike protein (anti-S) (Table S1). Over two-thirds of those enrolled exhibited no evidence of previous COVID-19 infection at the time of vaccination (*n* = 70, 68.6%) (i.e., COVID-19-naïve, CN). Others were deemed COVID-19-experienced (CE) if any one of these was positive, except for one individual (Subject #41) who self-reported a positive RT-qPCR test but had no symptoms nor any detectable antibody response in monthly samples tested before and after the RT-qPCR test, and thus was categorized as naïve with a presumed false-positive RT-qPCR test. Of the 32 individuals defined as CE, 19 met all three of the criteria above, 8 met two of the criteria, and 5 met one of these criteria (Table S1). Two subjects defined as CE (#17 and #22) were anti-S-negative prior to vaccination but had other documentation of infection. Subject #17 had multiple RT-qPCR-documented COVID-19 infections pre-vaccination with severe symptoms each time, but did not generate a detectable antibody response after these infections. However, this subject responded robustly to the first dose of vaccine, consistent with a memory response. Subject #22 became anti-N positive after their first dose of vaccine, and thus appears to have become infected during the vaccination process. The characteristics of the CE and CN participants were similar except for higher rates of self-reported cardiovascular disease (21.4% vs. 6.3%; *p* = 0.06) and slightly more healthcare workers (90.0% vs. 78.1%, *p* = 0.13) in the CN cohort compared to CE (Table 1).

### 3.2. Antibody Responses to SARS-CoV-2 mRNA Vaccination

**Figure 1:**
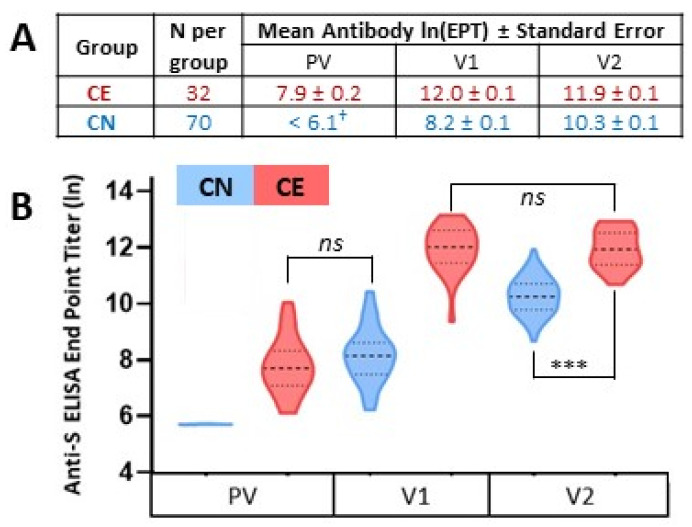
Antibody responses to SARS-CoV-2 mRNA vaccination quantified by anti-spike ELISA. Serially diluted serum samples were subject to hexapro antigen-based ELISA to determine the dilution at which positive antigen-binding was lost, that is, the endpoint titer (EPT). (**A**) The natural log of the mean EPT ± standard error of COVID-19-experienced (CE; red) and COVID-19-naïve (CN; blue) groups were calculated for samples obtained pre-vaccination (PV), 3 weeks after the first vaccine dose (V1), and 3 weeks after the second vaccine dose (V2). (**B**) The natural log of the mean and quartiles are graphed (y-axis). † The lowest antibody dilution tested was 1:450; hence, negative samples were defined as <1:450 and are graphed at a titer of 1:450 (i.e., ln 6.1). ns = not significant. *** represents *p* < 0.001. Two-sample *t*-tests were performed for comparisons of CN to CE. Paired *t*-tests were used for within-group comparisons (e.g., CE from V1 to V2).

A standard measure of vaccine response is the level of target antigen-specific antibodies detectable in the serum. Therefore, we measured anti-S antibody levels in longitudinal serum samples in persons with and without prior SARS-CoV-2 infection. Levels were quantified by end-point dilution ELISA. Prior to vaccination, COVID-19-naïve participants had levels of antibody binding to the full-length extracellular domain of the SARS-CoV-2 spike protein (i.e., hexapro) similar to pre-pandemic negative controls, and were therefore designated as seronegative (Figure 1, CN-PV, blue). After the first vaccine dose, all previous seronegative participants exhibited an anti-S response above the pre-pandemic controls; however, the levels were variable (Figure 1, CN-V1, blue). Upon second antigen exposure with the second vaccine dose, the COVID-19-naïve group exhibited an average 6.4-fold increase in anti-S levels and there was less variability in the antibody levels (Figure 1, CN-V2, blue). Consistent with this, there was an inverse relationship between CN-V1 antibody levels and the level of boost observed in response to the second dose of vaccine (R^2^ = 0.403; *p* < 0.001) (Figure S1A). In the CE group, all but two individuals had detectable levels of anti-S pre-vaccination with the range and variability of antibody levels being similar to that observed in the CN group after their first antigen exposure via vaccination (*p* = 0.06) (Figure 1, CE-PV, red). As a group, these pre-existing, infection-induced antibody levels were increased almost 50-fold after the first dose of vaccine, with this subsequent exposure resulting in a more uniform antibody level within the group (Figure 1, CE-V1, red). This pattern is similar to that observed in the CN group upon the second antigen exposure. Again, consistent with the more uniform antibody level after this second antigen exposure, there was an inverse relationship between the PV antibody levels and the level of antibody boost observed in response to the first dose of vaccine in the CE group (R^2^ = 0.409; *p* < 0.001) (Figure S1B). However, in contrast to the pattern observed in the CN group, the CE group exhibited no additional increase in antibody levels following the second vaccine dose (*p* = 0.77) (Figure 1, CE-V2, red). Regardless, there was a significant difference in the final antibody levels achieved in the CN and CE groups, with the CE group reaching higher antibody titers (*p* < 0.0001).

**Figure 2:**
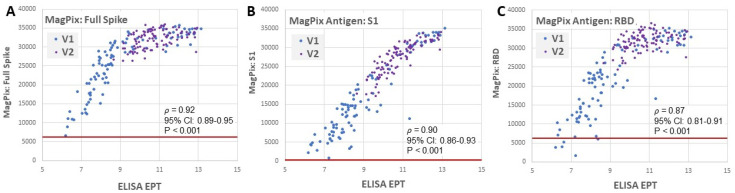
Correlation of anti-spike ELISA with MagPix immunoassays to different spike regions. Multiplex immunoassays were performed using recombinant antigens containing different regions of the viral spike proteins: (**A**) SARS-CoV-2 full trimeric spike, (**B**) the spike S1 domain, and (**C**) the spike RBD. Graphed is the Magpix median fluorescence intensity for each bead set (y-axis) and corresponding ELISA endpoint titer (x-axis). The red line represents the MagPix 98% cutoff values for each antigen: full spike = 6364, S1 = 337, RBD = 6360. Spearman’s rank correlation coefficient with 95% confidence intervals and *p*-value are shown.

Because antibody detection is dependent on the antigen bait used in individual assays, we performed a second, independent assay to assess reactivity against other spike antigens. We used the MagPix multiplexed immunoassay to measure serum reactivity against an analogous full-length spike antigen, the spike S1 domain, and the spike RBD. Relative binding to all three of these antigens correlated well with the initial ELISA results except that these assays demonstrated saturation at higher antibody levels as endpoint dilution was not performed (Figure 2A–C). In all cases, reactivity against the S1 and RBD of Spike was observed after the full two-dose vaccination was completed; however, five CN individuals did not exhibit anti-RBD binding after a single dose of vaccine (Figure 2C).

### 3.3. Factors Associated with the Magnitude of Vaccine-Induced Antibody Response

Because prior SARS-CoV-2 infection has a profound effect on vaccine response and notable variability in vaccine response was observed in the CN group upon initial exposure to the first dose of vaccine (Figure 1), our efforts to identify host factors associated with vaccine response focused on the CN group, whose first exposure was a controlled antigen dose administered at a known time. We utilized linear regression modeling to determine if specific host factors correlate with the magnitude of the vaccine-induced antibody response in COVID-19-naïve individuals (Table 2). In contrast to previous reports that older age is associated with higher antibody responses to SARS-CoV-2 infection [40,41], subjects aged 50 years and older exhibited lower antibody levels throughout the vaccination process (*p* = 0.01). However, further analysis revealed that this was driven primarily by the significant difference observed after the first vaccine dose (8.4 ± 0.1 among < 50 vs. 7.7 ± 0.1 among ≥50 years old; *p* < 0.001). Non-Caucasians exhibited higher antibody endpoint titer (EPT) after both the first vaccine dose (8.7 ± 0.2 vs. 8.0 ± 0.1; *p* = 0.005) and the second vaccine dose (10.7 ± 0.2 vs. 10.2 ± 0.1; *p* = 0.03). Consistent with a prior report [42], antibody EPT was higher in those who received the Moderna vaccine compared to Pfizer (*p* = 0.05); however, we observed that it was the response to the second dose that exhibited the most significant difference between the two mRNA vaccines (10.8 ± 0.2 vs. 10.2 ± 0.1; *p* = 0.004). The five CN individuals who did not demonstrate reactivity to the spike RBD after the first vaccination dose all received the Pfizer vaccine, but the differences in percent non-reactivity were not significant (0% Moderna vs. 8.8% Pfizer (*p* = 0.58)). However, analogous to the hexapro ELISA response levels (Table 2), those who received the Moderna vaccine had higher RBD-binding after their first dose of vaccine (Moderna (21,577 ± 1966) compared to Pfizer (16,698 ± 7407) (*p* = 0.03)). In agreement with recent reports [32,33], we saw no overall differences in vaccine antibody response between males and females (*p* = 0.33). However, analysis of the levels achieved after the first vaccine dose revealed a higher response in females (*p* = 0.01) that was not maintained after the second vaccine dose (*p* = 0.45). Co-morbidities, which are often found to be colinear with age, exhibited a similar pattern with a difference in levels observed after the first vaccine dose (*p* = 0.03), but in the end had no effect on the final V2 outcomes (*p* = 0.34).

**Figure 3:**
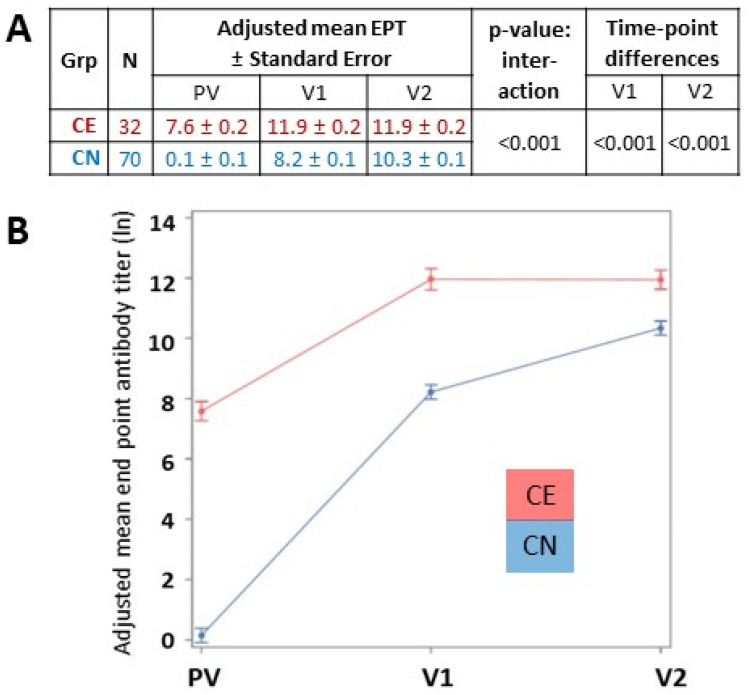
Differences in vaccine response between CE and CN individuals controlling for age, gender, and race. (**A**) The natural log of the adjusted mean antibody EPT ± standard error of COVID-19-experienced (CE; red) and COVID-19-naïve (CN; blue) groups was calculated for samples obtained pre-vaccination (PV), 3 weeks after the first vaccine dose (V1), and 3 weeks after the second vaccine dose (V2). (**B**) The natural log of the mean ±95% confidence interval is graphed (y-axis).

We next examined the differences in antibody titers observed between the CN and CE groups while controlling for covariates including age, gender, and race. This analysis confirmed that the adjusted antibody EPT varies significantly by COVID-19 experience over time (Figure 3). At each time point, the COVID-19-experienced group had a higher adjusted antibody EPT (*p* < 0.001 for all comparisons). Analysis controlling for vaccine type (i.e., Moderna vs. Pfizer) was also performed, but the three-way interaction was not significant (*p* = 0.92) (Figure S2) and so the simpler model without vaccine type is presented.

The magnitude of vaccine response was compared for each participant characteristic using a separate linear mixed model that included the characteristic of interest, time, and an interaction term. Adjusted mean EPT ± standard error was calculated for each subgroup at each time, and the significance of differences was assessed at V1 and V2.

### 3.4. Relationship between Serum Antibody Levels and Virus Neutralization

**Figure 4:**
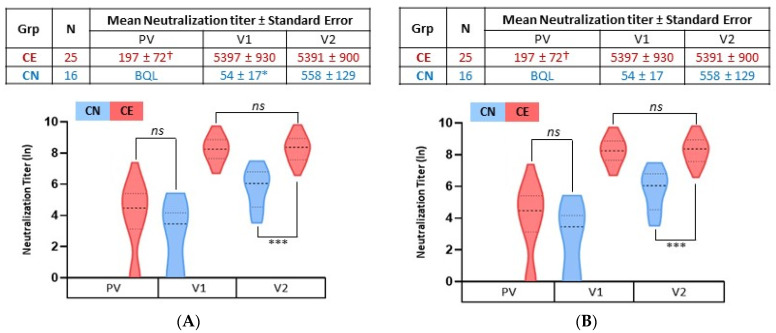
SARS-CoV-2 neutralization titer after mRNA vaccination. Serially diluted serum samples were subject to an infectious SARS-CoV-2 microneutralization assay to determine the dilution at which virus neutralization was lost. (**A**) The mean neutralization titer (NT) ± standard error of COVID-19-experienced (CE; red) and COVID-19-naïve (CN; blue) groups were calculated for samples obtained pre-vaccination (PV), 3 weeks after the first vaccine dose (V1), and 3 weeks after the second vaccine dose (V2). (**B**) The natural log of the mean NT and quartiles are graphed (y-axis). † Samples BQL were assigned a value of 1 in calculation of means and for ln transformation in the graph. ns = not significant. *** represents *p* < 0.001. *p < 0.01.

**Figure 5:**
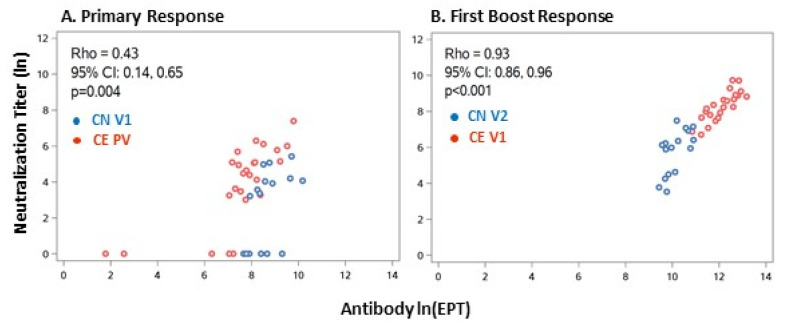
Comparison of antibody EPT and neutralization titer. (**A**) Primary response. (**B**) First boost response. Natural log of antibody EPT (*x*-axis) graphed against natural log of NT (*y*-axis). Spearman’s rank correlation coefficient with 95% confidence interval for antibody EPT and NT was calculated in the overall sample at each response time-point.

Several previously published anti-SARS-CoV-2 antibody titers quantified by ELISAs have been shown to correlate with neutralizing titers [13,43,44]. To determine whether our anti-S measurements corresponded with virus neutralization, we assessed antibody neutralization of a subset of samples using a microneutralization assay that tests the ability of the sera samples to neutralize infectious SARS-CoV-2 [13]. We assayed samples from 25 of the 32 COVID-19-experienced participants for which we had the necessary V1 samples and 16 random samples from the 70 COVID-19-naïve participants. Overall, the neutralization titers show a similar pattern to that observed for the antibody EPT (Figure 4). As expected, prior to vaccination, COVID-19-naïve participants had no neutralization activity (Figure 4, CN-PV, blue). After the first vaccine dose, only 10 of the 16 CN participants exhibited neutralizing activity, even though all 16 had detectable anti-S antibodies. As a group, the CN exhibited a relatively low mean neutralization titer (54 ± 17) (Figure 4, CN-V1, blue). While anti-S levels were similar after initial antigen exposure regardless of source (i.e., infection or vaccination) (Figure 1), neutralization titer tended to be lower after a single dose of vaccine in the CN group compared to the pre-vaccination titers observed in the CE group; however, the levels were not found to be statistically different (*p* = 0.08) (Figure 4, CE-PV vs. CN-V1). Upon second antigen exposure with the vaccine, all those in the CN group mounted a neutralizing response (Figure 4, CN-V2, blue). In the CE group, 20 of 25 individuals had detectable but variable neutralization activity prior to vaccination (197 ± 72) (Figure 4, CE-PV, red). As a group, this pre-existing, infection-induced neutralization activity was increased 27-fold after the first dose of vaccine. As observed for antibody levels, the CE group exhibited no additional increase in neutralization titer following the second vaccine dose (*p* = 0.20) (Figure 4, CE-V2, red). Analogous with the antibody data, there was also a significant difference in the final neutralization titer achieved in the CN and CE groups, with the CE group reaching a 9.7-fold higher mean neutralization titer (558 ± 129 vs. 5391 ± 900) (*p* < 0.0001). Direct comparison of the antibody EPT and neutralization titer revealed a significant but not perfect correlation (Figure 5). In particular, there were nine individuals (six CN; three CE) who developed antibodies after their first antigen exposure but failed to exhibit detectable virus neutralization (Figure 5A). There was insufficient power to determine whether any particular participant characteristic was associated with lack of neutralization activity, but all were female (*p* = 0.11), all were healthcare workers (*p* = 0.35), and all received the Pfizer vaccine (*p* = 0.20). The correlation between antibody level and neutralization titer was stronger after the second exposure (Rho = 0.93, 95% CI 0.86–0.96) (Figure 5B). These results highlight that additional vaccine doses may be required to elicit a robust neutralizing antibody response in some individuals.

## 4. Discussion

Serological assays are essential tools in the management of infectious diseases and assessment of vaccine response. In agreement with accumulating reports, we demonstrated that the two SARS-CoV-2 mRNA vaccines, BNT162b2 (Pfizer–BioNTech) and mRNA-1273 (Moderna), induce robust antibody responses to full-length spike, S1, and the RBD. We report that in our cohort, a single exposure to antigens, either via infection (CE PV) or via vaccination (CN V1), induces a variable antibody response which is affected by age, gender, race, and co-morbidities. Consistent with previous reports [45,46,47,48,49,50], the patients who had experienced COVID-19 exhibited a boost response to the first vaccine dose, but had no significant increase in antibody or neutralization titer in response to the second vaccine dose. Utilizing a hexapro-based ELISA, we showed that COVID-19-naïve individuals who received the Moderna vaccine exhibited a more robust boost response to the second vaccine dose (*p* = 0.004). Importantly, as observed with other published ELISAs [13,43,44], our hexapro-based assay antibody results correlate well with infectious SARS-CoV-2 neutralization titers.

Clinical studies have raised the question of the utility of vaccinating CE individuals within the first 6 months after infection, as infection rates and hospitalizations in CE individuals have been observed to be lower than in vaccinated, CN persons [51,52]. Our data emphasize that at least one vaccine dose in CE individuals increases antibody and neutralization titers by 50- and 27-fold, respectively (Figure 1 and Figure 4), that a second antigen exposure is required to elicit a detectable neutralization titer in some individuals (Figure 4), and that it greatly improves the correlation between antibody levels and neutralization (Figure 5). While our study was not designed to correlate immune responses with protection from infection or hospitalization, this at least suggests that vaccination of CE individuals may enhance protection and allow for more reliable monitoring via antibody testing. Because anti-S antibody levels did not increase significantly in the CE group after the second vaccine dose, the other question that has been raised is whether a second vaccine dose in this population offers any additional benefit [46,53,54,55,56]. Our data are consistent with a lack of increase in antibody level and neutralization titer following a second vaccine dose in CE people. However, the second dose in recovered individuals may have other immunological effects (i.e., increasing the durability of response, enhancing T cell response, broadening of the antibody response). Indeed, recent studies suggest that vaccine boosting after natural infection and after initial vaccination in CN individuals leads to better neutralization capacity to SARS-CoV-2 variants of concern [28,49,57]. Importantly, we hope to address issues of durability through continued monitoring of the current cohort.

The difference in vaccine response between COVID-19-naïve and COVID-19-experienced individuals has been reported previously; however, it is important to note that regardless of whether the initial exposure is from natural infection or vaccination, the antibody levels detected following the initial exposure in the two groups are similar (Figure 1**,** CE-PV compared to CN-V1). This is consistent with a recent report that compared serial samples from 10 CE and 207 CN individuals [53]. Although many variables dictate the response measured in the CE-PV group, the dose and timing of the first antigen exposure is well-controlled in the COVID-19-naïve group, suggesting that host factors are likely driving the observed variability. While others have shown that disease severity and the time post-infection correlate with differences in antibody levels post-SARS-CoV-2 infection, the similar antibody level and degree of variability we observed in the CE-PV and CN-V1 groups suggests that even in the context of infection, a large portion of the variability in the primary antibody response to infection is driven by host factors.

The tendency of host factors (e.g., age, gender, race, and co-morbidities) to correlate specifically with the initial vaccine antibody response in the CN group is interesting. This may be related to the molecular differences between an initial immune response versus a memory response, that is, perhaps these variables only impact the immune pathways involved in initial immune responses and not in memory responses. Alternatively, it is possible that this association disappears after the first dose because the majority of individuals are achieving their maximal response potential and are thus hitting some sort of biological immune response limitation. This could also explain the lack of increased antibodies observed in the CE group after the second vaccine dose. Monitoring the response of this cohort to additional booster vaccine doses may provide further insight into this question.

While our collective understanding of the immune responses to SARS-CoV-2 infection and vaccination is advancing rapidly, defining what is needed for protection, including target antibody levels, remains a challenge [58]. Importantly, our data provide further evidence that serum antibodies can be utilized as a reasonable surrogate for neutralization, particularly after a second antigen exposure. However, it would be informative to figure out why this correlation appears to fail in a small percentage of cases after the initial antigen exposure. Of the 198 samples for which antibody and neutralization titer were compared, nine (5%) exhibited no detectable neutralization despite robust antibody levels. This has important implications for assessing functional vaccine response based on antibody levels.

Limitations of this study include the relatively small sample size and the predominance of women and healthcare workers. However, it contributes to the growing data needed to answer critical questions about how infection- and vaccine-induced immunity contributes to the control of SARS-CoV-2 and provides insight into the differences and similarities in responses induced by infection versus vaccination, and between COVID-19-naïve versus COVID-19-experienced individuals. Ongoing collection of samples from this cohort will further our understanding of the immune response to booster vaccinations and the durability of the antibody response.

## Figures and Tables

**Table 1:** Participant characteristics by COVID-19 experience.

	Overall	COVID-19-Experienced	COVID-19-Naïve	*p*-Value
	*N* = 102	*N* = 32	*N* = 70	
Age, mean (SD) (*n* = 100) *	46 (13)	46 (15)	46 (12)	0.81
**Female, *n* (%)**	77 (75.5)	23 (71.9)	54 (77.1)	0.57
**Race/ethnicity, *n* (%)**				
Caucasian	79 (77.5)	22 (68.8)	57 (81.4)	0.52
Hispanic	8 (7.8)	3 (9.4)	5 (7.1)	
Black	4 (3.9)	2 (6.3)	2 (2.9)	
Asian	9 (8.8)	4 (12.5)	5 (7.1)	
Other	2 (2.0)	1 (3.1)	1 (1.4)	
**Healthcare worker, *n* (%)**	88 (86.3)	25 (78.1)	63 (90.0)	0.13
**Self-Reported Comorbidities, *n* (%)**				
Diabetes	4 (3.9)	1 (3.1)	3 (4.3)	0.99
Cardiovascular disease	17 (16.7)	2 (6.3)	15 (21.4)	0.06
Immunocompromised	1 (1.0)	0 (0.0)	1 (1.4)	0.99
Lung disease	6 (5.9)	2 (6.3)	4 (5.7)	0.99
Other	2 (2.0)	1 (3.1)	1 (1.4)	0.53
None	77 (75.5)	27 (84.4)	50 (71.4)	0.16
Vaccine received **, *n* (%)				
Pfizer	81 (79.4)	24 (75.0)	57 (81.4)	0.46
Moderna	21 (20.6)	8 (25.0)	13 (18.6)	

Patient characteristics are presented overall and stratified based on previous COVID-19 infection (experienced versus naïve). Group differences were assessed for statistical significance using a *t*-test for age and chi-square or Fisher’s exact test for nominal variables. * Overall participant number was 102, except for age as two participants did not provide their age. ** All participants received the same vaccine brand for both their first and second doses.

**Table 2:** Characteristics associated with the antibody response in the CN population.

	*N*	Adjusted Mean Antibody Ln (EPT) ± Standard Error			*p*-Value: Interaction	Time-Point Differences	
		PV	V1	V2		V1	V2
**Age**							
<50	41	0.1 ± 0.1	8.4 ± 0.1	10.4 ± 0.1	0.01	<0.001	0.19
≥50	28	0.1 ± 0.1	7.7 ± 0.1	10.1 ± 0.1			
**Gender**							
Female	54	0.1 ± 0.1	8.3 ± 0.1	10.3 ± 0.1	0.33	0.01	0.45
Male	16	0.0 ± 0.2	7.8 ± 0.2	10.2 ± 0.2			
**Race/ethnicity**							
Caucasian	57	0.1 ± 0.1	8.0 ± 0.1	10.2 ± 0.1	0.045	0.005	0.03
Non-Caucasian	13	0.0 ± 0.2	8.7 ± 0.2	10.7 ± 0.2			
**Comorbidities**							
Any	20	0.1 ± 0.1	7.9±0.2	10.1±0.2	0.50	0.03	0.34
None	50	0.0 ± 0.2	8.3 ± 0.1	10.3 ± 0.1			
**Vaccine received**							
Pfizer	57	0.1 ± 0.1	8.1 ± 0.1	10.2 ± 0.1	0.048	0.05	0.004
Moderna	13	0.0 ± 0.2	8.5 ± 0.2	10.8 ± 0.2			

Separate linear mixed models regressed antibody ln (EPT) on each participant characteristic, time, and an interaction term to estimate adjusted means.

## Data Availability

The data presented in this study are provided de-identify herein. Additional information may be available via data usage agreement on request from the corresponding author.

## Supplementary Materials

The following supporting information can be downloaded at: https://www.mdpi.com/article/10.3390/v14020370/s1. Figure S1: Inverse relationship between pre-existing antibody levels and fold increase observed after subsequent antigen exposure; Figure S2: Linear mixed effects models regressed the natural log of the endpoint antibody titer value on COVID-19 experience, vaccine type, and time; Table S1: Demographics and COVID-19 experience of Participants.
