# Supplementary material for: Antibody Response to SARS-CoV-2 Infection and Vaccination in COVID-19-naïve and Experienced Individuals
Source: Viruses. 2022 Feb 10;14(2):370. doi: 10.3390/v14020370 (PMC8878640; doi:10.3390/v14020370)
Supplement: Supplementary file 1 [file viruses-14-00370-s001.zip › Figure S1 with legend.pdf]

Supplementary Figure S1

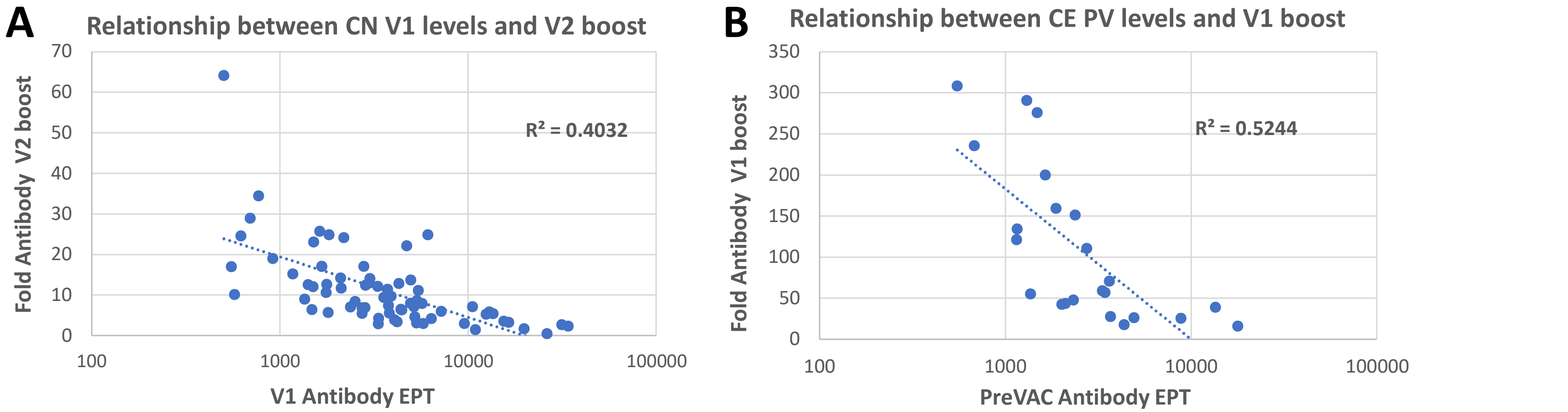

Figure S1. Inverse relationship between pre-existing antibody levels and fold increase observed after subsequent antigen exposure. Antibody EPT (x-axis) graphed against fold boost increase (y-axis). (A) Analysis of boost response in CN individuals in response to their second vaccine dose. (B) Analysis of boost response in CE individuals in response to their first vaccine dose.
