# Supplementary material for: Antibody Response to SARS-CoV-2 Infection and Vaccination in COVID-19-naïve and Experienced Individuals
Source: Viruses. 2022 Feb 10;14(2):370. doi: 10.3390/v14020370 (PMC8878640; doi:10.3390/v14020370)
Supplement: Supplementary file 1 [file viruses-14-00370-s001.zip › Figure S2 with legend.pdf]

Supplementary Figure S2

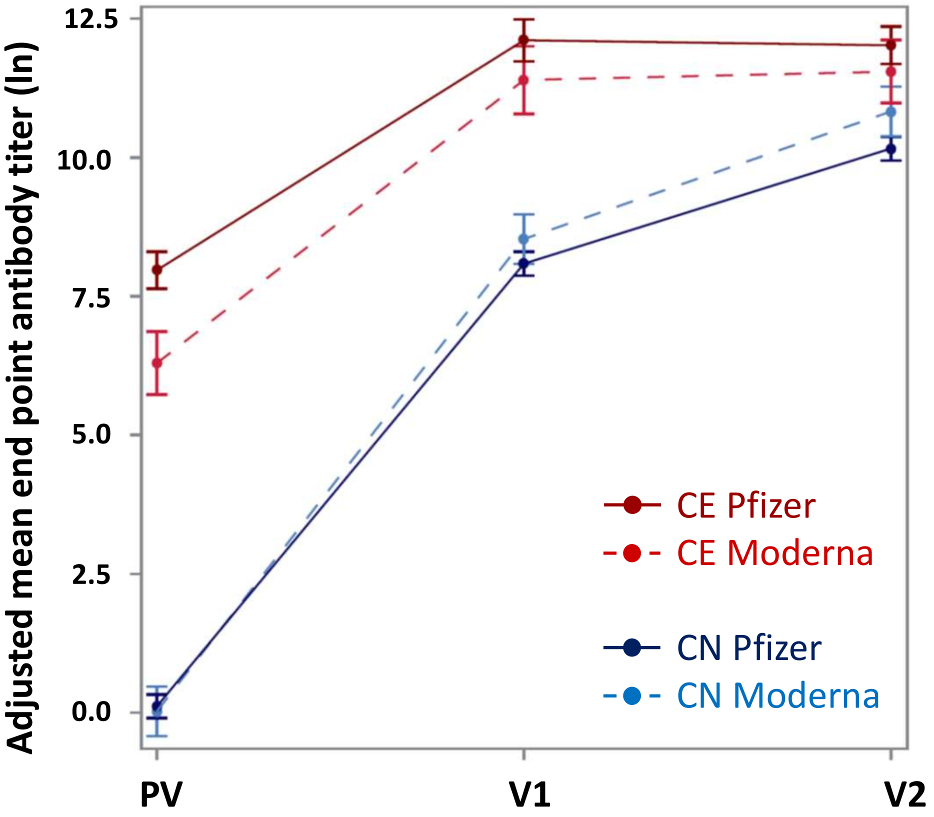


**Figure S2.** A linear mixed effects models regressed the natural log of the endpoint antibody titer value on CoVID-19 experience, vaccine type, and time, all two-way and one three-way interaction, and included age and gender as adjustment variables. The three-way interaction was not significant (p=0.92) and so a simpler model without vaccine type were presented in the main text.
