# Supplementary material for: Antibody Response to SARS-CoV-2 Infection and Vaccination in COVID-19-naïve and Experienced Individuals
Source: Viruses. 2022 Feb 10;14(2):370. doi: 10.3390/v14020370 (PMC8878640; doi:10.3390/v14020370)
Supplement: Supplementary file 1 [file viruses-14-00370-s001.zip › Table S1.pdf]

Subject	Gender	Age	Race	HCW	Vaccine	RT-qPCR	PV anti-N	PV anti-S ELISA	Group
1	M	63	A	Y	P	P	P	P	**CE**
2	F	48	C	Y	P	P	P	P	**CE**
3	F	35	C	Y	P	P	P	P	**CE**
4	F	26	C	Y	P	P	N	P	**CE**
5	F	63	C	Y	P	ND	N	P	**CE**
6	M	46	C	Y	P	ND	P	P	**CE**
7	F	49	A	Y	M	ND	P	P	**CE**
8	F	34	A	Y	P	P	P	P	**CE**
9	F	26	H	Y	P	P	P	P	**CE**
10	M	67	C	N	P	P	P	P	**CE**
11	F	34	C	Y	P	ND	P	P	**CE**
12	F	43	M	Y	M	P	P	P	**CE**
13	F	21	B	N	M	P	P	P	**CE**
14	M	43	H	N	P	P	N	P	**CE**
15	F	67	C	Y	P	P	P	P	**CE**
16	F	65	C	Y	P	P	P	P	**CE**
17	F	56	C	Y	M	P	P*	N	**CE**
18	F	65	C	N	M	P	P	P	**CE**
19	F	65	C	N	P	P	P	P	**CE**
20	F	X	C	N	P	ND	N	P	**CE**
21	F	57	C	N	P	P	P	P	**CE**
22	F	29	C	Y	M	ND	P*	N	**CE**
23	F	63	C	Y	P	P	P	P	**CE**
24	F	44	C	Y	P	P	P	P	**CE**
25	M	53	C	Y	P	P	P	P	**CE**
26	M	62	B	Y	P	ND	P	P	**CE**
27	F	44	H	Y	P	P	P	P	**CE**
28	M	32	C	Y	M	P	P	P	**CE**
29	M	29	C	Y	P	ND	P	P	**CE**
30	M	47	C	Y	P	ND	N	P	**CE**
31	F	29	C	Y	M	P	P	P	**CE**
32	F	28	A	Y	P	ND	N	P	**CE**
33	F	36	C	Y	P	ND	N	N	**CN**
34	F	52	C	Y	P	ND	N	N	**CN**
35	F	43	A	Y	P	ND	N	N	**CN**
36	F	33	C	Y	P	ND	N	N	**CN**
37	F	41	C	Y	P	ND	N	N	**CN**
38	F	36	C	Y	P	ND	N	N	**CN**
39	M	61	C	Y	P	ND	N	N	**CN**
40	F	38	C	Y	P	ND	N	N	**CN**
41	F	44	C	Y	P	P**	N	N	**CN**
42	F	52	C	Y	P	ND	N	N	**CN**
43	F	51	C	Y	P	ND	N	N	**CN**
44	F	33	C	Y	P	ND	N	N	**CN**
45	M	24	C	Y	P	ND	N	N	**CN**
46	F	63	C	Y	P	ND	N	N	**CN**
47	M	43	C	Y	P	ND	N	N	**CN**
48	F	27	C	Y	P	ND	N	N	**CN**
49	F	40	C	Y	P	ND	N	N	**CN**
50	M	41	C	Y	M	ND	N	N	**CN**
51	M	52	C	Y	P	ND	N	N	**CN**
52	F	50	C	Y	P	ND	N	N	**CN**
53	F	49	B	Y	M	ND	N	N	**CN**
54	F	59	H	Y	P	ND	N	N	**CN**
55	F	65	C	Y	P	ND	N	N	**CN**
56	F	25	C	Y	P	ND	N	N	**CN**
57	M	60	C	Y	P	ND	N	N	**CN**
58	F	47	C	Y	P	ND	N	N	**CN**
59	F	30	H	Y	P	ND	N	N	**CN**
60	F	28	H	Y	P	ND	N	N	**CN**
61	F	39	C	Y	P	ND	N	N	**CN**
62	F	61	C	Y	M	ND	N	N	**CN**
63	F	54	C	Y	M	ND	N	P	**CN**
64	F	62	C	Y	M	ND	N	N	**CN**
65	F	29	H	Y	M	ND	N	N	**CN**
66	M	45	C	Y	P	ND	N	N	**CN**
67	M	63	C	Y	P	ND	N	N	**CN**
68	F	44	A	N	P	ND	N	N	**CN**
69	F	48	C	Y	M	ND	N	N	**CN**
70	F	50	C	Y	P	ND	N	N	**CN**
71	F	52	H	Y	P	ND	N	N	**CN**
72	F	57	C	Y	P	ND	N	N	**CN**
73	F	57	C	Y	M	ND	N	N	**CN**
74	M	39	C	Y	P	ND	N	N	**CN**
75	F	39	O	Y	P	ND	N	N	**CN**
76	F	51	C	Y	P	ND	N	N	**CN**
77	F	33	C	Y	P	ND	N	N	**CN**
78	F	63	C	Y	P	ND	N	N	**CN**
79	F	53	C	N	P	ND	N	N	**CN**
80	F	37	C	Y	P	ND	N	N	**CN**
81	F	69	C	N	M	ND	N	N	**CN**
82	F	41	A	Y	P	ND	N	N	**CN**
83	F	36	A	Y	P	ND	N	N	**CN**
84	F	47	C	Y	P	ND	N	N	**CN**
85	F	40	B	Y	P	ND	N	N	**CN**
86	M	27	C	Y	P	ND	N	N	**CN**
87	M	51	C	Y	P	ND	N	N	**CN**
88	M	33	C	Y	P	ND	N	N	**CN**
89	F	42	C	Y	P	ND	N	N	**CN**
90	F	49	C	Y	P	ND	N	N	**CN**
91	F	68	C	N	M	ND	N	N	**CN**
92	F	x	C	Y	P	ND	N	N	**CN**
93	F	40	C	Y	P	ND	N	N	**CN**
94	M	60	C	Y	P	ND	N	N	**CN**
95	M	65	C	N	P	ND	N	N	**CN**
96	M	36	C	Y	P	ND	N	N	**CN**
97	F	38	C	Y	M	ND	N	N	**CN**
98	F	61	C	N	P	ND	N	N	**CN**
99	F	25	A	Y	P	ND	P/N***	N	**CN**
100	M	54	C	N	P	ND	N	N	**CN**
101	F	35	C	Y	M	ND	N	N	**CN**
102	F	28	C	Y	M	ND	N	N	**CN**

Table S1. Demographics and CoVID-19 experience of Participants. RT-qPCR was self-reported. Anti-N was measured by two assays: Abbot and MagPix. Anti-S was measured by Hexapro-based ELISA. Abbreviations: A=Asian; C=Caucasian; H=Hispanic; B=Black; M-mixed. P=positive; N=negative; ND=not determined. CE=CoVID-19 experienced; CN=CoVID-19 naive. No one reported a new RT-qPCR positive test result during the course of the study.

*Anti-N positivity was measured in the V1 sample.
**Self-reported RT-qPCR judge to be false-positive.
***MagPix anti-N results was negative. Abbot Anti-N was positive. However, Abbot signal appeared to be cross-reactivity as it did not decline in over time.
